# Developing composite indices of geographical access and need for nursing home care in Ireland using multiple criteria decision analysis

**DOI:** 10.12688/hrbopenres.13045.1

**Published:** 2020-09-16

**Authors:** Brian P. Reddy, Stephen O'Neill, Ciaran O'Neill

**Affiliations:** 1 JE Cairnes School of Business and Economics, National University of Ireland, Galway, Galway, Ireland; 2 Patient Access Services, Novartis, Dublin, Ireland; 3 London School of Hygiene & Tropical Medicine, London, UK; 4 School of Medicine, Dentistry and Biomedical Sciences, Queen's University Belfast, Belfast, UK

**Keywords:** nursing homes, older adults, multiple criteria decision analysis, composite index

## Abstract

**Background:** Spatial accessibility has consistently been shown to influence utilisation of care and health outcomes, compared against local population needs. We sought to identify how appropriately nursing homes (NHs) are distributed in Ireland, as its NH market lacks central planning.

**Methods:** We used multiple criteria decision analysis (MCDA) approaches to develop composite indices of both access (incorporating measures of availability, choice, quality and affordability) and local NH need for over 65s (relating to the proportion living alone, with cognitive disabilities or with low self-rated health, estimated scores for activities of daily living and instrumental activities of daily living, the average number of disabilities per person and the average age of this group). Data for need were derived from census data. Results were mapped to better understand underlying geographical patterns.

**Results:** By comparing local accessibility and need, underserved areas could be identified, which were clustered particularly in the country’s northwest. Suburbs, particularly around Dublin, were by this measure relatively overserved.

**Conclusions: **We have developed multi-dimensional indices of both accessibility to, and need for, nursing home care. This was carried out by combining granular, open data sources and elicited expert/stakeholder opinion from practitioners. Mapping these data helped to highlight clear evidence of inequitable variation in nursing home distribution.

## Introduction

A decline in the availability of informal care for older people in Ireland by families and church groups must either be filled by government and/or the private sector formal care ^1^ or else go unmet. The nursing home (NH) market has grown steadily in Ireland since the 1980s. Means-tested subsidies for care were theoretically available from government over this period, but did not always see provision of nursing home beds where needed. Blanket tax incentives to build new private nursing homes were offered in the early 2000s in an attempt to increase supply ^2^ but without direction as to where to build these did not address local supply issues. Overall there was essentially an absence of a coherent policy on the part of government to a growing need for formal residential care in Ireland over this period ^3^.

Since 2009 the “Health Information and Quality Authority” (HIQA) has been responsible for inspecting and registering care homes. Public nursing homes are operated by the “Health Service Executive” (HSE) - a publicly funded state agency that provides care. Private NHs are owned by a variety of for- and not-for-profit operators, with an increasing presence of international groups ^2^. Private homes dominate the market, making up about 79% of homes and 81% of beds. A government scheme named the “Fair Deal” subsidises the payments individuals must pay for NH care: residents pay a proportion of their income and a further proportion of other financial assets (such as savings, shares or property); the government pays the remainder through a body known as the National Treatment Purchasing Fund (NTPF).

Across health sectors, accessibility has consistently been shown to influence utilisation of care ^4, 5^ and health outcomes ^6– 8^. Hart’s inverse care law states that access to medical care tends to vary inversely with the need for it in the population served, particularly in private markets ^9^. Given the predominant role of the private sector in Irish NH care, this paper aims to provide an exploratory examination of whether local NH accessibility is appropriate to local needs in Ireland.

As both need and access, as defined in this paper, are multidimensional we created composite indices to reflect each of these concepts using a multi criteria decision analysis (MCDA) approach to aggregate and to explore the relationships between them.

MCDA techniques can be used to better structure complex decision problems ^10^, increase transparency ^11^ and avoid the problems associated with using heuristic approaches to solve decision problems involving complex trade-offs ^12^. MCDA approaches have been used in a wide range of health settings and their use has grown in recent years ^13, 14^. They are considered ideally suited to the creation of composite indices ^15^, including in health settings ^16^.

McIntyre *et al*. ^17^ group determinants of accessibility into three broad dimensions: availability (whether appropriate services are available where and when they are needed), affordability (the ability to pay and consideration of the opportunity costs of doing so) and acceptability (cultural perspectives/conditions that empower patients to use services and to ‘fit’ with provider attitudes). The World Health Organisation essentially groups them into availability, affordability and the need for “information accessibility”: the “right to seek, receive and impart information and ideas concerning health issues” ^18^.

Whereas access is inextricably linked to where the homes are physically located (amongst other factors such as the capacity of homes), the level of need for NH care in a location relates to its underlying population characteristics and demographics.

The remainder of this paper is set out as follows. Section 2 describes how each step of the MCDA process was approached in the creation of the indices. Section 3 describes an overview of the results, highlighting patterns of need, access and access-need ratios. Section 4 provides a discussion of how well services are aligned to needs, implications of the results, and limitations of the process used to develop the indices. Section 5 presents the study’s conclusions.

## Methods

There are many MCDA methods. These fall into the broad groups of value measurement, outranking and goal programming ^19^. No single approach is perfect for every decision problem, and choice of MCDA method should be based on resource/time constraints, scientific validity and the significance and broader context of the decision problem ^11^.

Regardless of the approach used, there are a commonly agreed set of steps required in applying MCDA techniques. This section is laid out following the steps described in Marsh *et al.* ^20^ (other very similar schemas exist ^21^). These steps, described below in the indicated sections, were: ‘defining the decision problem’; ‘selecting and structuring criteria’ and ‘measuring performance’; ‘scoring alternatives’; ‘weighting criteria’; ‘calculating aggregate scores’ and ‘dealing with uncertainty’ (incorporating sensitivity analyses); and ‘reporting and examining of findings’.

A standard linear additive MCDA model was chosen as the most appropriate approach given the aims of this paper and the data available. In this approach, a simple weighted sum of normalised scores on each criterion is calculated to create a composite “overall score” for each location. This is the most commonly used approach in healthcare ^13^ and has the advantages of being relatively simple to understand compared to other MCDA techniques, being easy to compute and to compare results. This simplicity is particularly important given the relatively large number of geographical units in this study and the aim to create composite indices. Alternative, non-compensatory MCDA approaches, including outranking and goal programming approaches, focus on issues such as dominance or satisficing, which would lack meaningfulness in this context, particularly as comparisons of cardinal scores would not be possible.

There are various ways to weight criteria, and swing weighting is the most commonly used for this approach, as it allows for trade-offs between dimensions (i.e. performing poorly on one criterion can be made up for by good performance on others) and because it fulfils the theoretical requirements of MCDA ^19, 22^.

### Defining the decision problem

This stage requires understanding the background to the problem, triangulating clearly articulated aims, generating a list of stakeholders that could be invited to participate, and deciding on which steps they will be required to take part. The balancing of both scientific/technical and social/human factors - referred to as a “socio-technical” approach in the literature ^21^ - can be something of an art, but must be considered as part of this process. The paper describes a process whose primary purpose was as an evidence or knowledge generating exercise, and stakeholders invited to take part were acting as independent experts who could share their experience. MCDA approaches are particularly well suited to converting such expert experience into quantifiable scores ^11^.

### Selecting criteria and measuring performance

There are a number of factors to consider in choosing criteria for an MCDA model ^20^:

- Completeness: criteria should capture all relevant factors for the decision.
- Non-redundancy: criteria should be removed if irrelevant.
- Non-overlap: Criteria should be defined so that they avoid “double counting”, where the same effect is counted in more than one criterion, leading to it receiving more weight than is intended.
- Preference independence: The criteria can be analysed one at a time, and performance on one does not depend on performance on another.

These are guiding principles rather than hard rules, and as elsewhere any final model remains a simplification. Estimates of performance on each of the criteria can be gathered in a variety of ways, ranging from evidence synthesis to expert elicitation where relevant quantifiable data are unavailable ^11^.

Accessibility-related criteria were calculated by location, while need was estimated using secondary data from the national census. Expert opinion was therefore only required in weighting the criteria and in helping to interpret the results.

***Access.*** Data used for calculating accessibility came from combining several standalone datasets. A list of all registered NHs nationally is available from the HIQA website ^23^, which was downloaded in June 2019. The geolocation of many homes was provided therein; others were calculated manually by inputting the associated address into Google Maps. Data from HIQA inspection reports (covering the period Feb 2018-May 2019) were used to measure NH care quality. Prior to February 2018, a separate inspection regimen was used, which would have otherwise made prior comparisons challenging. Fair Deal fees per resident were calculated using a dataset provided by the NTPF, covering the period January 2019 – September 2019. Such data on fees was not available from the homes that are operated by the HSE.

We defined four types of accessibility relevant to nursing home care that broadly correspond to headings described by McIntyre and the WHO described in the *Introduction*, reflecting: the availability of nearby services (availability), the range of choice of NHs (acceptability), the NTPF fee paid for those services (affordability), and a simple measure of NH quality based upon HIQA inspection reports (information). Their formal definitions are presented below. Each was based upon relationships between NH locations and each electoral district’s (ED) centroid. EDs are the smallest legally defined administrative areas in the State, allowing the greatest possible granularity for our results. A total of 3,409 EDs were included, with their boundary files being downloaded from the Central Statistics Office (CSO) website. The four criteria used to build a composite measure of “overall accessibility” were:

- *Availability:* Spatial accessibility to care, based upon a gravity potential model ^24^. This is the most sensitive approach for explaining population access to services ^25^, allowing all homes to be considered and weighted by their number of beds, whether inside a given ED’s boundaries or not. The availability score for each ED *i* is calculated using the formula $\sum_{j}[(n_{j}/\max(d_{ij}^{2},1))],$ where *n _j_* is the number of beds in each nursing home *j* and *d _ij_* is the distance in kilometres between the centroid of ED *i* to nursing home *j*. Distances less than 1km were set to this level to avoid attaching disproportionate weight to NHs close to the ED centroid (resulting from calculating $1/d_{ij}^{2}$ ).
- *Consumer choice:* This referred to local competition level in the NH market, using the Herfindahl-Hirschman Index (HHI). This reflects the market share of homes in each market, weighted by their number of beds. We selected a 12.5 km radius as relevant, as in Grabowski ^26^, weighting by the number of beds in each home. Markets with a HHI <0.15 are considered competitive, between 0.15-0.25 are moderately concentrated, and between 0.25-1 are highly concentrated ^27^.
- *Affordability:* The estimated average weekly fee per bed within a 12.5-km radius of the ED centroid. If there were no homes within this radius, the average by county was used. Note that this measure is based upon private homes’ fees only; the fees of HSE-run homes were not available. Because fees to privately funded residents are not available, we assume Fair Deal fees to be an indicative proxy of private fees (given Fair Deal fees in homes were themselves originally based upon the fees charged to private patients there).
- *Quality:* Distance to the nearest “fully compliant” home, which we defined as an NH where – in its most recent HIQA inspection – all criteria that were tested were either compliant or substantially compliant. This included only NHs that have been inspected since the current testing protocol began in Spring 2018.

The indicators for these four criteria are used as a basis for aggregating into a composite index of overall accessibility at ED level nationally.

***Need.*** As part of a scoping exercise, a targeted literature search was carried out to identify factors that influence need for nursing home care. The search string ("nursing home*" OR "elderly care home*") AND TOPIC: (admission* OR admit*) AND TOPIC: (predict*) was used, with the last search conducted on 12 December 2019. Papers describing the risk of admission related to patients with specific, single issues for which we did not have access to data are not included (these included falls, delirium and Parkinson’s disease). Six papers ^28– 33^ were identified from a search of “Web of Science” that used meta-analyses, literature reviews or multinomial models to identify factors that were associated with nursing home admission. All six were US-based. Several of the papers described factors in terms of the influential “Behavioural Model of Health Services Use” model headings ^34^, which describes the drivers of accessibility and healthcare utilisation in terms of predisposing factors, enabling factors and need. They are reported using this model in Table 1.

**Table 1.** Overview of factors found to be significant predictors of nursing home admission from prior literature. +, positive relationship; -, negative relationship; ns, non-significant. For Luppa, only factors strong or moderate evidence are included in this table.

	Gaugler	Miller	Lo Sasso	Guralnik	Greene	Luppa*	Sum	Included
Predisposing								
Demographic								
Age	+	+	+	+	+	+	6	✓
White race	+	+	+		+	+	5	
Female	-	ns	ns	+	ns		2	✓
Social support								
Lives alone	+	+			+		3	✓
Available caregiver	+	-			ns		2	
Greater familial support		-	-				2	
Married	-	ns			ns		1	
Number of children	-		ns				1	
Spouse present	-						1	
Widowed			+				1	
Enabling								
Familial resources								
Homeowner	-	-			-	-	4	✓
Low/missing income	+	ns			ns		1	
Unemployed						+	1	
Wealth		ns	ns				0	
Education	ns	ns	ns		ns		0	
Market/policy resources								
Bed supply		+			+		2	
Urban		ns			ns		0	
Need								
Self-perceived								
Self-reported poor health			+		ns	+	2	✓
Life satisfaction	ns				ns		0	
Practitioner evaluated								
Physical function								
Activities of daily living (ADL)	+	+	+	+	+	+	6	✓
Instrumental activities of daily living (IADL)	ns	+	+		+	+	4	✓
Cognitive function								
Cognitive impairment	+	+	+		+	+	5	✓
Disease								
Dementia/Alzheimer’s dx		+				+	2	
Diabetes	+	ns					1	
Cancer	+						1	
Stroke	+						1	
Hypertension		-					1	
Muscular-skeletal		-					1	
Nervous/sense		+					1	
Depression/mental		+					1	
Digestive		-					1	
Number/severity of illness		+					1	✓
Other health issues								
Greater social activity		-				-	2	
Falls	+	ns					1	
Needs help climbing stairs				+			1	
Need help walking half a mile				+			1	
Low activity level						+	1	
Catheter					ns		0	
IV tubes					ns		0	
Use								
Prior nursing home use	+	+				+	3	
Prior hospitalisation	+	+					2	
Number of medications		+				+	2	
Paid helper		+					1	
Physician visits					-		1	

This created a longlist of variables for potential inclusion. To be included in our final MCDA need model, variables further had to be judged to be relevant in an Irish context and thereafter to be mappable to relevant data from the most recent (2016) census. Similar approaches using census data that have been reported elsewhere ^35, 36^. have noted the utility of such open data approaches in developing a composite index for public use. After liaising with the CSO, we gained access to summaries of data confined to populations over 65. However, these could only be made available at the level of 31 geographical units (rather than the 3,409 units at ED level) for data privacy reasons. These corresponded with either county or city borders and, for convenience, we will refer to these throughout the paper as ‘counties’.

Where possible, direct or plausible proxy data were chosen which reflected these variables of need. These were not always perfect: our ADL measure for example excluded certain aspects such as toileting, which is not recorded in the census. Other promising candidate variables, such as social activity levels and prior nursing home admission, were excluded entirely as relevant data was not available.

Gender’s impact upon need for care appeared to be inconclusive varying between studies. We assumed that having a male majority meant lower need before talking to stakeholders, with scope to change this depending on their views and experience. The proportion that are “white American” was not felt to be relevant in an Irish nursing home setting and was not included in the model shown to participants. The formal definitions used for each variable, its median and the location of its maximum and minimum scores are shown in Table 2.

**Table 2.** Data used for each variable representing need, alongside its minimum, median and maximum score and associated location.

Factor	Data used	Min	Median	Max
Age	Average age of over 65s (all respondents over 100 or over assumed to be 100 years old).	73.15 (South Dublin)	74.14	75.10 (Dublin City)
Living alone	Proportion of over 65s in private housing living alone	0.205 (Fingal)	0.269	0.327 (Leitrim)
Home not owner occupied	Proportion of over 65s not in housing owned (with/without mortgage, and ignoring those who did not answer this question)	0.081 (Meath)	0.111	0.180 (Cork City)
Cognitive disability	Proportion of over 65s with “Difficulty in learning, remembering or concentrating”	0.044 (Co Cork)	0.051	0.068 (Dublin City)
Activities of daily living	Proportion of over 65s with difficulty dressing, bathing or getting around the home	0.070 (Fingal)	0.091	0.103 (Dublin City)
Average disabilities per person	Average number of disabilities per person over 65	0.825 (Fingal)	0.980	1.205 (Dublin City)
Instrumental activities of daily living	Average (for over 65s) of number of issues of • Going outside the home to go shopping or visit a doctor • Working at a job or business, or attending school or college • Participating in other activities, such as leisure or using transport	0.267 (Fingal)	0.333	0.394 (Dublin City)
Low self **-**rated health	Proportion of over 65s who regarded themselves as having bad or very bad health.	0.039 (Co Cork)	0.051	0.072 (Dublin City)
Gender	Proportion of over 65s that are male	0.432 (Dublin City)	0.475	0.493 (Cavan)

### Scoring alternatives

Measurements were transformed using “value functions” which are used to ensure that scores on different criteria are normalised to a common 0-100 scale. Choosing an appropriate value function for a variable depends on the context of the data and relies somewhat upon the experience of decision makers and those designing the study. While value functions can be assumed to be linear for practical purposes in many cases ^21^, it is important that in principle that each point on the scale represents equal (within criterion) increments ^16^. Therefore, an ED scoring 50 on a criterion should be about halfway between the best performing and worst performing EDs on that criterion; if this lacks face validity then a linear value function would be unsuitable.

All functions for the value functions used are described in this section. Linear value functions were felt to be acceptable for most variables, including all of those related to need as they used a single, “per person” scale ^13^. All were assumed to follow a negative linear function (i.e. $\frac{\left(\max(x_{i})-x_{ij}\right)}{\left(\max(x_{i})-\min(x_{i})\right)}$ ), for criterion *i* and county *j*. Figure 2 shows the distribution of the need criteria after the application of these linear value functions, with 100 implying highest need and 0 the lowest. Certain geographical patterns are evident. For example, cognitive impairments rates are noticeably higher in Dublin than elsewhere, and need relating to ADLs is particularly high in the northern counties (alongside Dublin city centre).

 Linear value functions were also considered acceptable for two accessibility criteria (distance to quality homes and affordability), but for those relating to “choice” and “availability”, alternative value functions were required.

EDs with a HHI of 1 have a ‘choice’ of only one NH within the relevant radius. However, it was felt that the value function must further acknowledge that areas without any home in the given radius had, in some sense, even less choice. Such areas, with a HHI of “NA” were given the worst possible score on HHI of 0; the transformed HHI of other variables was calculated using the formula $\frac{e^{-HHI_{j}}}{e^{-\min\;(HHI)}},$ to ensure that the ED with the lowest HHI (and hence greatest competition) would itself be given a score of 1. This function meant that in practice EDs with access to a single home (and thus a 100% market share for a single home implying a HHI of 1) were given a score at approximately 0.373 on this criterion $(\text{i}.\text{e}.\frac{e^{-1}}{e^{-0.01442}}).$

Availability was highly skewed. It was felt that a diminishing marginal of utility of each additional bed was more plausible than each additional unit being of equal value. The accessibility score was therefore log transformed - thus reducing the impact of the most extreme upper values - and hence a positive linear transformation applied (i.e. an equation of $\frac{\log(max(x))-\log(x_{j})}{\log(max(x))-\text{log}(min(x))}$ )

**Figure 1.**
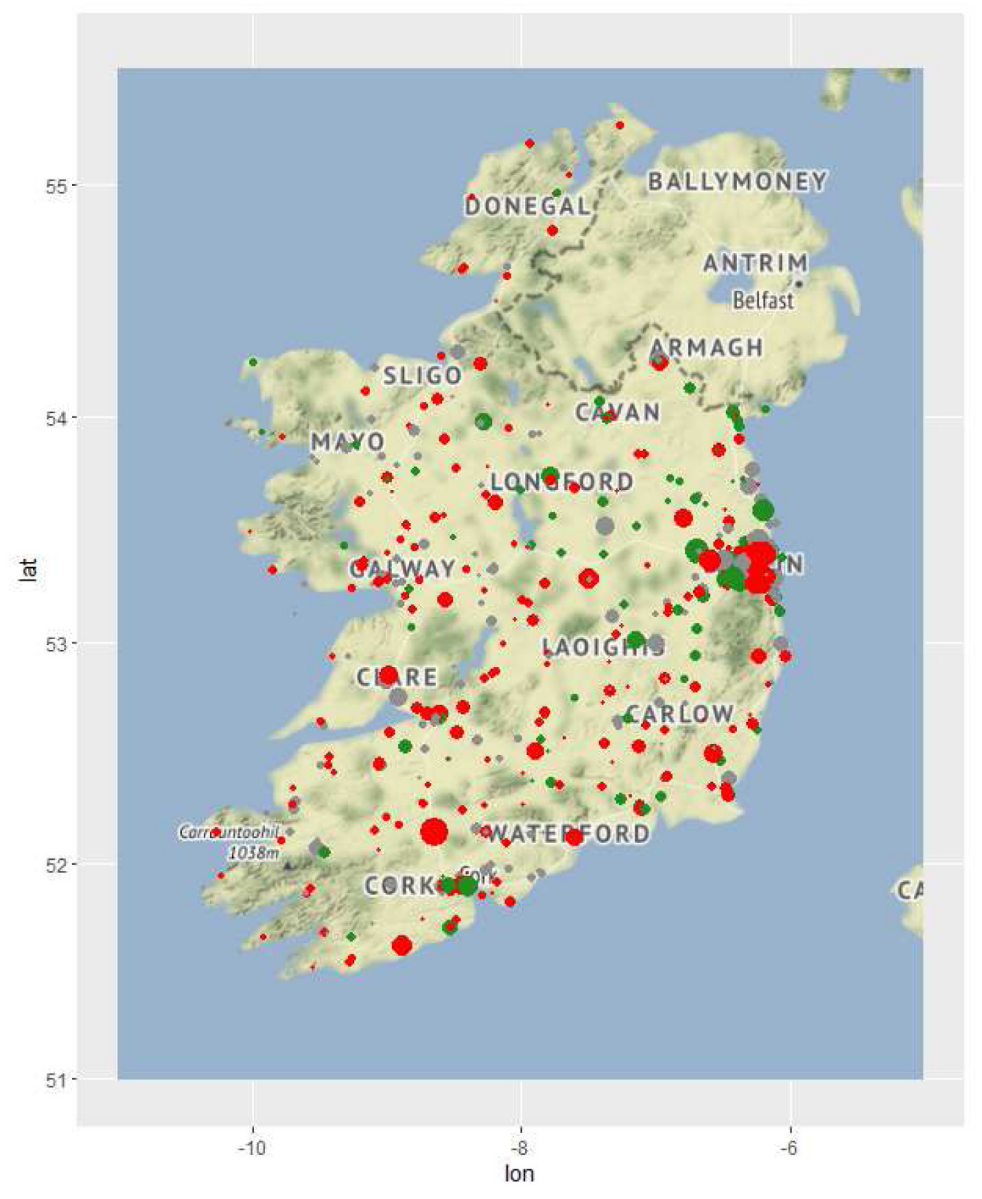
Nursing home locations, sized by number of beds and coloured by most recent inspection. Green, fully compliant; red, some criteria non-compliant; grey, no inspection over relevant period.

Figure 3 shows the distribution of the accessibility criteria after the application of the relevant value functions. It is clear, for example, that accessibility relating to affordability is particularly low in Dublin (i.e. fees are higher there), and that this gradually improves as EDs get further away. Fees are also generally relatively high near Cork City (in the south of the country) and in Sligo in the northwest. Areas of deep purple on the HHI map indicate EDs that have no homes within the 12.5-km radius. Availability is noticeably higher in Dublin than elsewhere. The minimum distance to a quality home is more dispersed around the country, which corresponds to the locations of homes marked green in Figure 1. 

**Figure 2.**
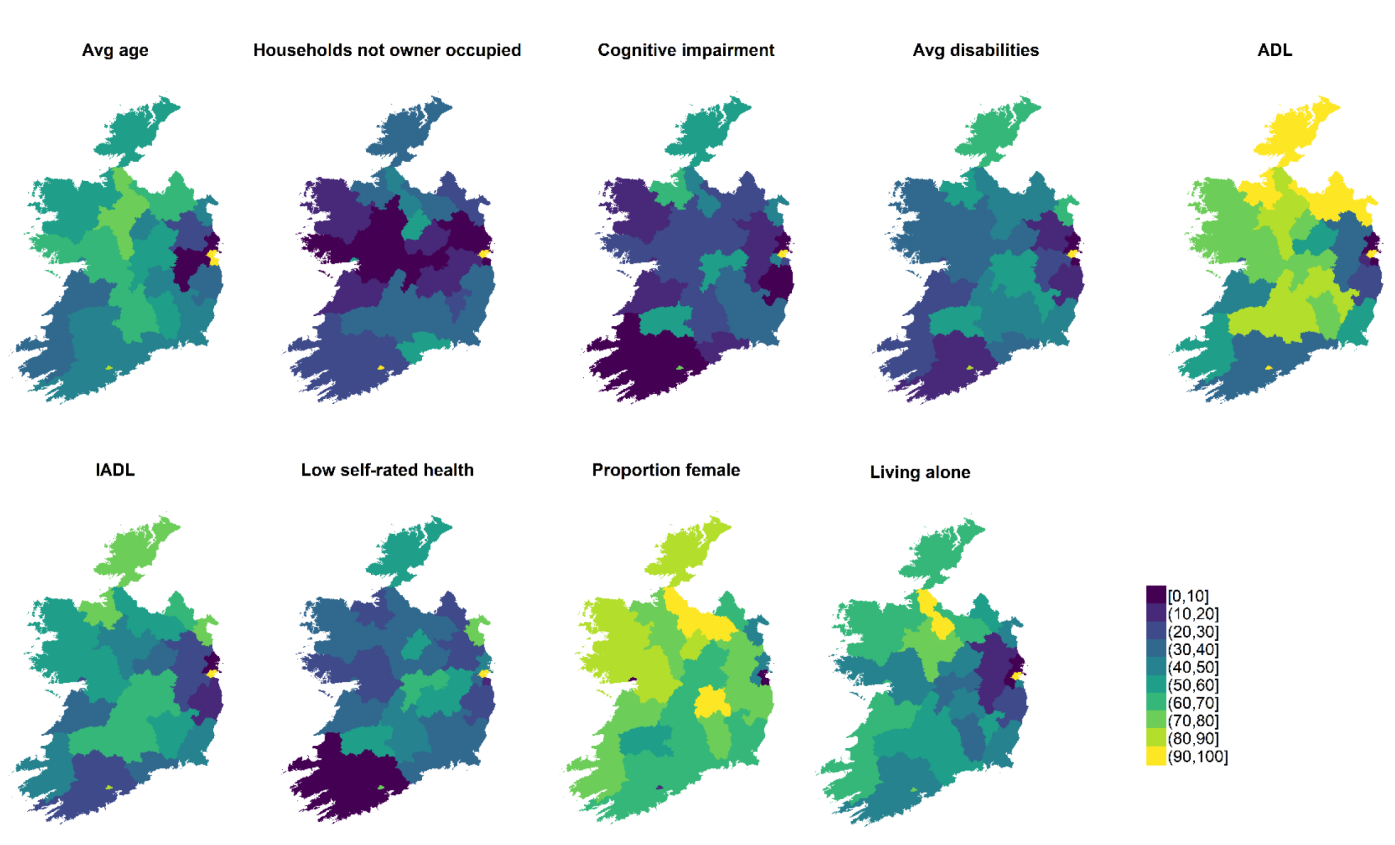
The nine crieria measuring nursing home need, after application of value functions. These are displayed by county, yellow in this case means highest need.

**Figure 3.**
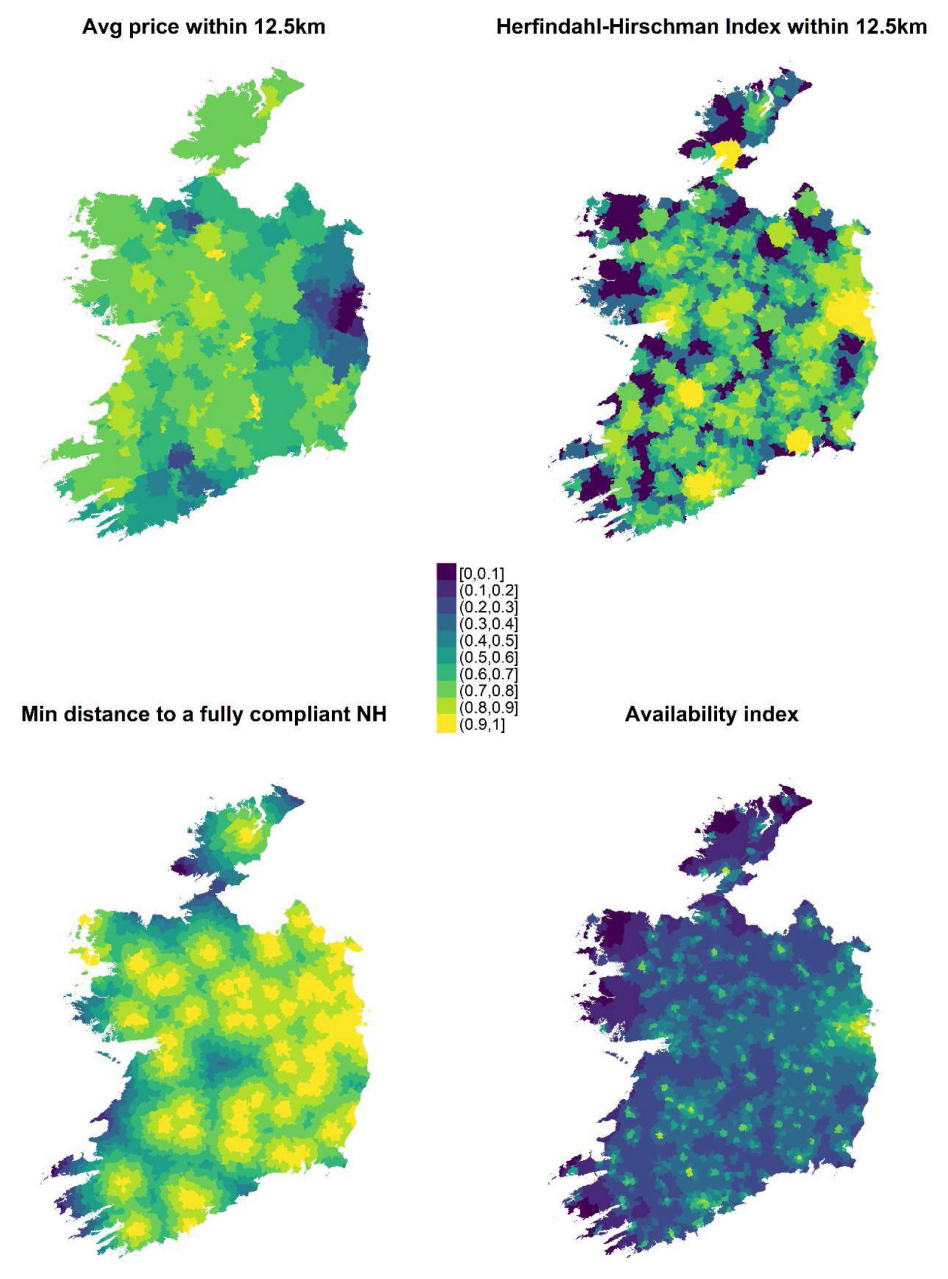
The four measures of accessibility to nursing homes, after the application of their value functions. They are displayed by ED, with purple indicating low access and yellow high access.

### Weighting criteria

Weighting is normally carried out by “stakeholders”, who have relevant expertise and are capable of expressing preferences and quantifying value judgements ^37^. Care must be used in selecting whose preferences are used ^37^ and the group must be large enough to be representative but small enough to be manageable and for decisions to be made within a reasonable timeframe ^38^. Our model uses the views of three individuals (two from non-governmental organisation ALONE (respectively the Head of Services and Projects Manager) and one from the NTPF (Contract Manager). ALONE is well placed to represent the views of older people, given its formal role as advocates for the group and its experience of matching individuals with NHs. The NTPF were approached to ensure that a payer perspective was also included.

Swing weighting requires that stakeholders explicitly consider only from the range the possibilities under consideration (from best performing action to worst, on each criterion) ^39^, rather than the feasible range. If all neighbourhoods had similar levels on a specific criterion, then its importance to the decision (and hence weighting) will be relatively small.

In the prior stage, all criteria were normalised on a 0–100 scale, employing suitable value functions to ensure internal consistency for each criterion in terms of utility. This weighting stage allows participants to ensure that the criteria can be scaled appropriately to reflect “how big is the difference… and how much do you care about that difference?” ^16^, with swing weights effectively operating as exchange rates between criteria. This allows a total overall score to thereafter be created using a weighted sum approach.

Participants are asked to imagine living in a neighbourhood with the worst score on all criteria and where there existed an opportunity to increase one criterion to match its ‘best’ score nationally. In this hypothetical scenario, whichever criterion they choose is thereafter considered the most important by the participant ^40^. Stakeholders are then asked which criterion they would change next, next after that, and so on. Each criterion is assigned a relative weight; for example, the swing for X is 80% as important as that of the most important criterion. These are thereafter collated and normalised. Individual and collated weights for both access and need are shown in Table 3.

**Table 3.** Calculation of swing weights for accessibility and need.

	Swing		Weights					
	Worst	Best	ALONE 1	ALONE 2	ALONE avg.	NTPF 1	Overall avg.	Norma-lised swing weights
**Accessibility**								
Average local bed fee (€)	1234	780	40	75	54.77	40	46.81	17.3%
Local choice of provider (HHI)	NA	0.04	20	40	28.28	80	47.57	17.6%
Distance to closest fully compliant home (km)	74.1	0.10	80	90	84.85	100	92.12	34.1%
Availability score	1	880	100	100	100	70	83.67	31.0%
**Need**								
Mean age	75.1	73.2	27.39	40.88	33.46	40	36.58	7.6%
Not owner occupied	0.18	0.08	0	48.27	0	50	0	0.0%
Cognitive disability	0.07	0.04	90	77.83	83.69	70	76.54	15.8%
Avg disabilities per person	1.2	0.8	85	63.05	73.21	80	76.53	15.8%
ADL	0.1	0.07	100	100	100	90	94.87	19.6%
IADL	0.39	0.27	52.92	92.61	70.00	100	83.67	17.3%
Low self-rated health	0.07	0.04	20	85.22	41.28	70	53.76	11.1%
Male	0.43	0.49	0	55.66	0	40	0	0.0%
Living alone	0.33	0.21	50.99	70.44	59.93	65	62.41	12.9%

***Access weights.*** An overall figure for ALONE was calculated using the geometric average of its two participants, and then the ALONE average was combined with the NPTF preferences again using the geometric average (with the two weighted equally) to create an overall average swing weight, which was subsequently normalised.

The ordering of preferences was identical for both ALONE participants, and quite similar for the NTPF representative. The weights are particularly dominated by the two criteria relating to distance – the nearby availability of beds and the distance to a fully compliant home. That distance to travel was found to be so important mirrors prior findings from Shugarman and Brown ^41^.

***Need weights.*** Criteria were initially ranked from most to least important by each stakeholder. For one participant from ALONE, initial rankings were thereafter transformed into weights using the procedure outlined by Alfares and Duffuaa ^42^, which she felt were an acceptable reflection of her preferences. The other ALONE participant developed her own weights based upon her initial rankings, but for certain criteria (mean age, IADL and living alone) preferred to represent these with a range rather than a point estimate. For the purposes of our baseline model, the geometric average of her upper and lower limits of the range were used; this is described further in *Sensitivity analyses results*. She also thought that the proportion by gender in the community and the proportion of homeowners were unlikely to be at all influential of need in her experience, and should be given a weighting of zero. These were similarly ranked lowly by both other participants, who were satisfied to use the geometric average again to collate a collective judgement. Again, overall the views of all participants are similar across criteria, described in sensitivity analyses. As previously, a collective ALONE judgement was combined with that of the NTPF representative (on a 50-50 basis), and swing weights were normalised.

### Calculating aggregate scores and incorporating uncertainty

Scores calculated for each geographical unit on each criterion were combined with the associated weight on each criterion to calculate overall accessibility scores for each electoral district, and overall need scores for each county. Both were performed using a standard weighted sum approach as shown below:

$$v_{j}=\sum_{0}^{i}s_{ij}\cdot w_{i}$$

where

- v _j_   is the overall score for county/ED *j* estimated from MCDA model
- s _ij_   is the score for geographical unit j on criterion *i*
- w _i_   is the weight attached to criterion *i*.

Each neighbourhood could thereafter be ranked by its overall score, whether for need or accessibility. Scores were subsequently mapped to identify geographical patterns in both accessibility and need, as well as the ‘ratios’ between these, which can highlight potentially underserved areas systematically. Such mapping was further carried out using cartograms that are warped by the local populations over 65, in order to better highlight if areas of high population are poorly served. Clustering within patterns is investigated and described, though some questions remain as to why certain areas remain underserved. Results of sensitivity analyses are presented in *Sensitivity analyses results*.

## Results

Urban areas were found to have particularly high levels of need, which is perhaps a counterintuitive finding. Dublin city was consistently amongst the highest need areas across criteria (as indicated in Table 2). Conversely, need is lowest in the counties surrounding urban areas, particularly around Dublin.

Accessibility is highest in the regional cities of Limerick and Waterford, which have relatively low fees for urban areas. Access is lowest in remote communities on the west coast for counties Donegal (in the north), and Kerry and Cork (in the south). Many of the least accessible EDs were in Irish language-speaking “Gaeltacht” areas.

**Figure 4.**
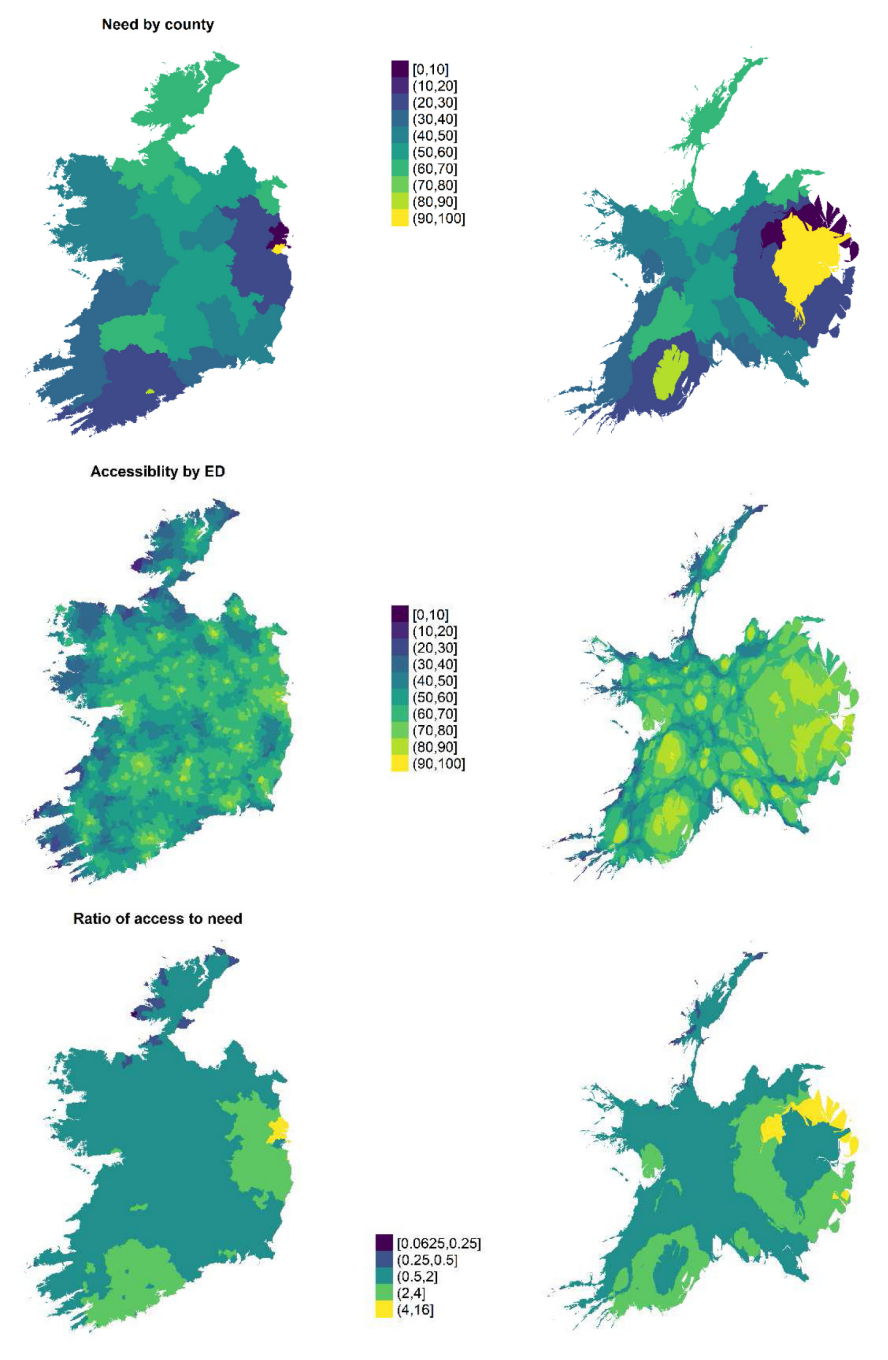
Need, accessibility and accessibility-need ratio (left) and corresponding cartograms warped by the size ED's population over 65 (right).

Figure 4 shows the overall need, accessibility and accessibility-need ratio maps (on the left), alongside cartograms which have been warped by the ED-level population of over 65s (on right). This paper’s composite index reflects a “place-based assessment” ^20^ and does not consider each district’s relevant population. This was a conscious decision to allow rates to be used to ease comparison during the scoring process ^18^. The cartograms highlight that given the concentration of need was found to be highest in urban areas, there are larger populations requiring NH care than might appear at first glance on the unwarped maps. There are also relatively large absolute populations of over 65s in commuter belts. The accessibility-need ratio map makes it apparent however that both the high levels of need in Dublin and Cork city centres appears to be somewhat met by high levels of access in the cities’ hinterlands.

All surrounding counties of Dublin have a better than expected access-need ratio, as do the rural parts of particular counties. On the other hand, distinct patterns exist in the relationships between city centres and rural hinterlands for other counties.

For the remainder of the country, the accessibility-need ratio is around one (i.e. between 0.5 and 2 on Figure 4), except in Donegal and Sligo in the northwest of the county (and isolated tips of peninsulas in Co Galway and Kerry). These areas have recurring issues of isolation and the problems associated with it, and again many are in Gaeltacht areas.

### Sensitivity analyses results

Baseline results were compared against recalculated results using:

- weights where all criteria were valued equally (for both accessibility and need);
- normalised weights based upon the highest valuation of in each of the range of ALONE2’s preferences; and
- normalised weights based upon the lowest weightings in each of the of the range of ALONE2’s preferences.

One often-used approach in MCDA to carry out sensitivity analyses is to calculate the changes in weightings required to change the final ranking of alternatives (i.e. neighbourhoods in our case). Such an approach is unsuitable in this case given the large number of neighbourhoods being compared – any perturbation whatsoever in the weightings would likely lead to some change in the ranking of EDs given there are 3,409 of them. Instead, Table 4 shows the correlations between the results of the baseline MCDA models reported previously, and the three alternative weighted models. They are extremely similar in all cases, and as a result the alternative models barely impact the results.

**Table 4.** Correlations between baseline model results and those of alternative models.

Correlation with:	Equal weighted version	Using ALONE 2 high weights	Using ALONE 2 Low weights
Baseline accessibility	0.9738	NA	NA
Baseline need	0.9915	0.9998	0.9999
Baseline ratio	0.9427	0.9978	0.9967

Correlations between rankings rather than scores are similar (and slightly higher), with the lowest such correlation being 0.9766 for the baseline ranking of need vs equal needs.

These results are not particularly surprising. It is a recurring theme in MCDA studies that despite the fact that weightings (and other stages) require fallible human judgements, findings of MCDA approaches are typically very robust ^43^.

*Underlying data* at nursing home-, district-, and county-level are available at Open Science Framework ^44^.

## Discussion

The aim of his paper was to develop univariate, composite indices of both accessibility and need for NH care in Ireland, and to investigate underlying patterning and relationships. The results reveal spatial variability in terms of both, most strikingly highlighting urban-suburban-rural divides. These differences are most clear in relation to need, and likely reflect the younger populations present in the commuter belts (the age profile of over 65s specifically were also clear in Figure 2). This may reflect the peculiar dynamics of the Irish housing market where a housing shortage forced younger persons in particular into suburbs by a shortage of suitable city centre accommodation. This was nonetheless a surprising finding as “urbanness” was found to be insignificant predictor of need for care in the (US-centric) literature review.

A recent study that developed a composite index of accessibility to health care in Germany found that city centres had the highest accessibility, and their donut-shaped hinterlands had the lowest ^45^. We similarly found that there was a clear relationship between the accessibility index and (logged) population density - a correlation 0.715. Indeed, the cartogram highlights that the cities never have low accessibility, and those areas with the lowest accessibility to care are sparsely populated. From a utilitarian perspective at least, this is somewhat consoling - though it is hardly reassuring to those living in poorly served areas.

The overall findings therefore were that urban areas have high accessibility but also the highest need, suburban areas have relatively high access but the lowest need, and rural areas typically have medium level of both access and need. Isolated areas of the western seaboard, particularly in counties Donegal and Sligo, are particularly underserved.

That Sligo has such poor accessibility is not helped by the relatively high fees for homes there – which are the highest outside of the greater Dublin area despite its relatively rural location. Nonetheless, this may be overstated as fee was considered the least important criterion (largely due to the existence of the Fair Deal scheme). If new homes were built there to take advantage of these high fees, this would most likely on balance increase overall accessibility in the county (through increases to the other criteria). That fees may poorly reflect the opportunity cost of land use though is possible.

As might be expected in circumstances where there is such a high level of private sector involvement in provision, the results seem to be in line with Hart’s inverse care law. Urban areas have particularly high need for homes but, likely due to the opportunity cost of property, relatively few NHs are located there. However, the large availability of homes in suburban hinterlands means that in absolute terms many new NH residents may not have far to travel *.* Whether or not the transportation of city centre residents away from their communities is acceptable is nonetheless a matter for debate; government policy ostensibly aims to allow patients insofar as possible to receive care within their own communities ^46^. The need for families to travel to visit relatives would be felt particularly acutely in deprived communities, which are particularly in city centres and rural areas – whereas well off suburban communities (coincidentally or otherwise) have the highest accessibility-need ratios. One ALONE participant felt strongly that income or deprivation levels should be incorporated in order to understand the impact on certain criteria relating to need; such data is not available from the census (and a deprivation score derived from it shows little variation at a county level).

While there were some differences between the weightings elicited from participants – such as the higher weighting that the NTPF participant placed upon the importance of competition compared to the ALONE stakeholders – overall the two groups agreed broadly on the importance and ranking of criteria. As might be expected, given the robustness of the results demonstrated in the sensitivity analyses, when the ALONE and NTPF models’ results are examined independently they are highly correlated: 0.9654 for accessibility and 0.9516 for need.

We also acknowledge a further complication in our approach: while accessibility fundamentally requires the consideration the availability of nursing home beds in the locality, such availability may induce demand. Indeed, bed supply was included as a potential cause of “need” in Table 1. A cardinal rule in MCDA models is the avoidance of double counting – whereby the same factor is included twice. If availability of beds was included as a factor on both sides of the access-need ratio, this could have undermined the interpretability or meaningfulness of findings. There is an inevitable trade-off in MCDA approaches between the need to be comprehensive and to keep models to a manageable size (and one which is comprehensible by stakeholders taking part), and no MCDA model can claim to be the final word on any issue as a result. We therefore concluded that bed availability should be confined to the accessibility side alone which, while imperfect, seemed to be the only justifiable way of dealing with this issue.

McIntyre’s acceptability of care reflects cultural issues and not all could be captured in our accessibility model. Currently there is no formal provision of NH care in minority languages. This is an issue which will likely need further consideration as the resident foreign-born population in Ireland ages. It is also clear that many of the worst served areas are in Gaeltacht areas where one assumes many of the residents have Irish as their first language, posing further difficulties given the vulnerability of elderly populations.

Ultimately the results of our approach can be considered a decision-making aid to provide insight to decision-makers, who can use their own judgement as to the most appropriate next steps. We might hope that new homes would be developed in the most underserved areas, which we interpret as those with high accessibility-need ratios. The government cannot compel private operators to build in such areas, however, though they do have levers such as targeted tax benefits to encourage provision or indeed the public provision of care to substitute for it. Either way there may be practical challenges in recruiting and retaining staff in such locations, given the low wages offered in the care sector and the high commuting costs to remote areas. As such, government interventions that provide grants to cover commuting/public transport costs or to help provide training in social care to local residents could be worth considering.

All parties in parliament have committed to supporting the “Sláintecare” plan for the health service which (amongst other issues) places emphasis on providing care in the community and increased home help hours. This may go some way to alleviate accessibility concerns of the most remote (as well as urban and other) communities. It may also prove to be a better use of resources than building and operating facilities in such places. It would be worthwhile for future researchers to consider the availability of home help, which currently varies around the country, and its impact on overall elderly care services.

### Limitations

Datasets had to be constructed and matched manually (due to the lack of a single unique identifier used across them) meaning that comprehensive and perfect datasets were not available. NH fees used related only to private NHs and, even then, only 424/460 private homes could be matched to a fee.

A further 195 homes not inspected over the relevant period could not be accredited as ‘fully compliant’ by our measure (and hence were considered as equivalent to non-compliant homes for this criterion). Of the remainder, 112 were fully compliant, and 274 not fully compliant.

Distance to travel used Haversine-, rather than road-distance, which may offer a better reflection of barriers to care and accessibility.

It was not possible to elicit views from representatives of the NH market to offer a provider perspective, which might have offered alternative viewpoints or helped to clarify why certain areas are underserved or seemingly unappealing for development.

Analyses were, as previously stated, constrained by the questions asked in the census. If other data sources had also been included it is possible that the need measure could have been more comprehensive. Notwithstanding this, we believe that the measure described in this paper is meaningful and requisite for describing the needs of local populations.

## Conclusions

We have used an MCDA approach to combine expert judgement, a targeted literature review and secondary data to create composite indices of nursing home accessibility and need, by geographical region in Ireland. Findings were found to be robust to alternative possible weighting approaches.

Composite indices of accessibility and need (and the ratio between them) have been calculated at a granular level across all EDs nationally. This paper has shown that there is variability in access and need across the country. Parts of the northwest of the country have the lowest ratio for accessibility to need and can therefore be considered to be underserved. Suburban areas have the highest such ratio.

Need for care for the average resident is highest in cities and lowest in suburban areas. Accessibility to NH care is highest in the smaller regional cities of Limerick and Waterford, and lowest along remote areas along the western seaboard.

The findings can be used as a practical decision-making aid to provide insight into future resource allocation decisions, by the government and other providers. The index used publicly available data and could be updated using future (or historical) census results.

Given that accessibility to health care has consistently been shown to influence healthcare utilisation, it is likely that areas with poor accessibility to NH care will utilise such care in inequitable and inefficient ways. If the government wishes to address such issues it will need to find ways to encourage the provision of new NHs along the western coast (and particularly in the north-western counties of Sligo and Donegal). It may also be worthwhile further considering how to better provide care within local communities, especially in remote underserved areas (and potentially in city centres where the need is highest). Further provision of home help services, perhaps targeted to such areas, may be part of this package.

## Data availability

### Underlying data

Open Science Framework: Developing composite indices of geographical access and need for nursing home care in Ireland, using multiple criteria decision analysis. https://doi.org/10.17605/OSF.IO/3DX2C ^44^.

This project contains the following underlying data:

- electoral_divisions.shp. (Geographical reference data allowing the electoral districts to be mapped.)
- Nursing home locations and details.csv. (Underlying data at nursing home level.)
- SAPS 2016 Glossary.xlsx
- Underlying needs and access for nursing home care by electoral district.csv. (Underlying data at electoral districts and county level.)

Data are available under the terms of the Creative Commons Attribution 4.0 International license (CC-BY 4.0).
