# Supplementary material for: Developing composite indices of geographical access and need for nursing home care in Ireland using multiple criteria decision analysis
Source: HRB Open Res. 2020 Sep 16;3:65. [Version 1] doi: 10.12688/hrbopenres.13045.1 (PMC8669779; doi:10.12688/hrbopenres.13045.1)
Supplement: Peer review history

# Peer review: Reviewer response for version 1

**DOI:** 10.21956/hrbopenres.14141.r30907

**Published:** 2021-12-13

**Authors:** Julien Mercille

**Affiliations:** 1 School of Geography, University College Dublin, Dublin, Ireland

This is an interesting paper that engages directly with a very important question, that of need and access in relation to nursing homes. The paper takes a geographical perspective to evaluate and try to quantify those metrics by area.

- One strength of the paper is that those questions are endlessly discussed in policy circles and so to bring a rigorous methodology to the issues is very valuable. However, the paper's quality overwhelmingly depends on the methodology, which I am not in a position to assess expertly. Therefore my report should be considered with that important caveat in mind.
- Other similar studies employing differing criteria would have to be conducted in order to assess which one is the "best" in relative terms. One avenue for further research that I could propose is to conduct a similar study but for a smaller geographical area (a county, or even a main town/city and surroundings). This would allow the authors to use their high-level methodology (as in this paper) but to tweak it based on precise knowledge of the area's needs, access problems, etc. This could highlight what criteria really matter for that area, and in what order of importance, etc. It would, I assume, help to understand the key obstacles to nursing home care in that area, which is ultimately one of the conclusions that is very important.

Is the work clearly and accurately presented and does it cite the current literature?

Yes

If applicable, is the statistical analysis and its interpretation appropriate?

I cannot comment. A qualified statistician is required.

Are all the source data underlying the results available to ensure full reproducibility?

Yes

Is the study design appropriate and is the work technically sound?

Yes

Are the conclusions drawn adequately supported by the results?

Yes

Are sufficient details of methods and analysis provided to allow replication by others?

Yes

Reviewer Expertise:

Privatisation and marketisation of care.

I confirm that I have read this submission and believe that I have an appropriate level of expertise to confirm that it is of an acceptable scientific standard.

# Peer review: Reviewer response for version 1

**DOI:** 10.21956/hrbopenres.14141.r27970

**Published:** 2020-11-26

**Authors:** Annabelle Glaize

**Affiliations:** 1 Management Department, LEM‐CNRS (UMR 9221), IÉSEG School of Management, Lille, France

**General comments:**

 This paper is a good and well structured application of an MCDA process. The context of application is pertinent and the authors worked hard in defining the criteria that would suit the decision problem, and so create a model as close to reality as possible. Various methods are used to propose a complete decision making process.

 The introduction should be developed a little bit to emphasize the need of the paper and the originality/adequacy of the methods chosen/used.

**Comments for the methods section:**

- Interesting to follow agreed set of steps for the structure of the paper. This part is well described in the method section.
- More details are needed as to the name of the MCDA method used (is it AHP?).
- Instead of saying that the method "is the most commonly used for this approach", I would suggest the authors to develop a little bit more the justification so it is clear that it is the best method. Otherwise, if the paper only uses "most commonly used methods", then what is the added value?

Is the work clearly and accurately presented and does it cite the current literature?

Yes

If applicable, is the statistical analysis and its interpretation appropriate?

Yes

Are all the source data underlying the results available to ensure full reproducibility?

Yes

Is the study design appropriate and is the work technically sound?

Yes

Are the conclusions drawn adequately supported by the results?

Yes

Are sufficient details of methods and analysis provided to allow replication by others?

Yes

Reviewer Expertise:

MCDA; health care

I confirm that I have read this submission and believe that I have an appropriate level of expertise to confirm that it is of an acceptable scientific standard.
